# Selective tau seeding assays and isoform-specific antibodies define neuroanatomic distribution of progressive supranuclear palsy pathology arising in Alzheimer’s disease

**DOI:** 10.1007/s00401-022-02480-x

**Published:** 2022-08-18

**Authors:** David G. Coughlin, Vanessa S. Goodwill, Heidi G. Standke, Yongya Kim, Nicolas Coley, Donald P. Pizzo, Douglas Galasko, Allison Kraus, Annie Hiniker

**Affiliations:** 1 Departments of Neurosciences, University of California, San Diego, USA; 2 Departments of Pathology, University of California San Diego, 9500 Gilman Drive, La Jolla, San Diego, CA 92093-0612 USA; 3 Department of Pathology, Case Western Reserve University School of Medicine, 2103 Cornell Road, Cleveland, OH 44106 USA

Incipient progressive supranuclear palsy (PSP) pathology in the setting of Alzheimer’s disease neuropathologic change (ADNC) has only rarely been reported (see Supplemental Table 1﻿ for key prior works) [1]. These works suggest that the stereotyped neuroanatomic distribution of AD-related 3R/4R tauopathy and PSP-related 4R tauopathy are preserved in this setting; however, this has not been rigorously examined. Recently, isoform-specific tau antibodies (RD3, RD4, and AD-specific GT38) and isoform-selective tau seeding assays (3R/4R and 4R real-time quaking-induced conversion [RT-QuIC]) have been developed by us and others (methods and references in Supplement) that we hypothesized should delineate the neuroanatomic localization of co-occurring 3R/4R and 4R-tau pathology. We tested this hypothesis in five cases of high ADNC with incidental PSP (“ADNC + PSPi”) pathology and found, first, that neuroanatomic distributions of 4R PSP and 3R/4R AD pathology are conserved in ADNC + PSPi and, second, that RT-QuIC can robustly distinguish the neuroanatomic distribution of 3R/4R versus 4R pathology in cases with mixed tau pathology, suggesting its utility as a tool to interrogate pathophysiology.

We reviewed 1296 autopsy cases with intermediate or high ADNC and identified 5 cases (0.3%) with incidental co-occurring PSP pathology (Table 1 for additional clinical and pathologic information). Age of onset was 72 ± 3.8 years and disease duration was 11 ± 2.1 years (mean ± SD). Two cases had mild parkinsonism; one had gait disorder 5 years after symptom onset. No cases met NINDS-SPSP or MDS criteria for possible or probable PSP [2]. Case 3 had extensive aging-related tau astrogliopathy (ARTAG, 4R tauopathy) throughout the brain.

**Table 1:** Clinical and neuropathologic characteristics of ADNC-PSPi cases

Case #	1	2	3	4	5
Clinical characteristics					
Sex	Female	Male	Male	Male	Female
Age of onset	67	70	76	74	75
Disease duration (y)	13	8	10	11	13
Clinical diagnosis	Probable AD	Probable DLB	Probable AD	Probable AD	Probable AD
NINDS-SPSP criteria	Not met	Not met	Not met	Not met	Not met
MDS-PSP criteria	a0, p0, a0, c0	a0, p0, a2, c2	a0, p0, a2, c0	a0, p0, a0, c0	a0, p0, a0, c0
APOE	3,3	3,4	3,3	3,4	3,3
Neuropathology					
Brain weight (g)	980	1236	1248	1158	1050
Thal phase	A2	A2	A2	A3	A3
Braak tau stage	VI, B3	VI, B3	V, B3	VI, B3	III, B2
CERAD stage	3	3	3	2	2
ADNC	Intermediate	Intermediate	Intermediate	High	Intermediate
LBD stage	None	None	None	None	Amygdala Predominant
Other	None	LATE Stage 2 with hippocampal sclerosis	ARTAG, LATE Stage 2 with hippocampal sclerosis	LATE Stage 2 with hippocampal sclerosis	LATE Stage 1 without hippocampal sclerosis

Immunohistochemistry and immunofluorescence for phospho-tau (AT8), 3R-tau (RD3), 4R-tau (RD4), and AD-specific tau (GT38) was performed on pons, midbrain, hippocampus, basal ganglia, temporal cortex, midfrontal cortex, and occipital cortex (see Supplement for methods). In ADNC + PSPi, we could distinguish AD pathology from PSP pathology by staining characteristics and morphology of inclusions: all pathological phospho-tau inclusions stained with AT8; AD neurofibrillary tangles (NFTs) but not PSP pathology stained with GT38 and RD3; PSP pathology stained with RD4 but not GT38. Consistent with the neuroanatomic distributions of each pathology in isolation, GT38-positive NFTs were abundant in midfrontal cortex and hippocampus, while RD4-positive astrocytes and oligodendroglia were prominent in midfrontal white matter and basal ganglia (Fig. 1a, S1). Semi-quantitative regional scoring demonstrated similar neuroanatomic distribution of AD versus PSP pathology in the five cases (Fig. 1b and Supplemental Results).

**Fig. 1:**
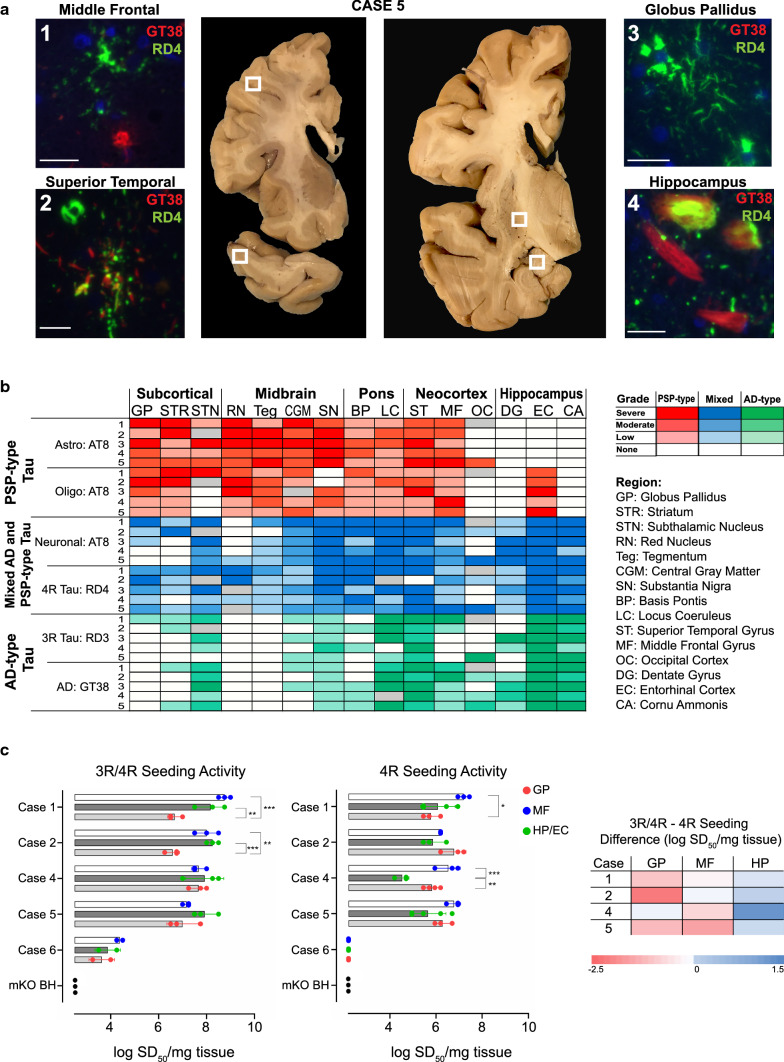
**A** Immunofluorescent profile of tau inclusions. Anatomic regions from Case 5 immunostained for GT38 (red), RD4 (green) and DAPI (blue) (Scale bars = 25 μm). **B** Regional distribution of tau pathology. Red: pure PSP-type tau glial pathology (AT8 +). Green: pure AD-type tau pathology (RD3 or GT38 +). Blue: mixed AD and PSP pathology. White: no pathology and grey: region unavailable. **C** RT-QuIC seeding activity**.** RT-QuIC analyses of 3R/4R and 4R seeding activity with heat map of differences in seeding activity by region and case (Controls: Case 6 = 35 year old Huntington's disease patient with Braak 0/VI and mouse tau knockout brain homogenate [mKO BH]). Seeding dose (SD_50_) is shown on a log_10_ scale (mean ± standard deviation), each data point is an independent replicate. Additional details in Supplement

To further discriminate neuroanatomic patterns of AD and PSP disease activity in these mixed cases, we performed 3R/4R and 4R RT-QuIC on regions with distinct tau isoform immunostaining: globus pallidus (high PSP, low AD pathology), hippocampus (low PSP, high AD pathology), and frontal lobe (high PSP, high AD pathology). Our published RT-QuIC methodology allowed us to quantify seeding activity over a large (10^9^-fold) dynamic range with regional seeding doses for 3R/4R-tau and 4R-tau consistent with semiquantitative immunostaining (Fig. 1c, S2-S4, Supplemental Results). Because Case 3 showed extensive ARTAG (4R-tau), it was excluded from RT-QuIC analysis. Importantly, seeding activity favored 4R-tau in the globus pallidus, 3R/4R-tau in the hippocampus, and was variable in the midfrontal cortex (*p* < 0.0001, see Supplement). Two cases of cognitively normal older adults (age of death: 75 and 84 years, both Braak stage I/VI) and two cases of Huntington’s disease (age of death: 35 and 37 years, both Braak stage 0/VI) were selected as controls. All four controls showed markedly lower 3R/4R and absent 4R seeding from all regions (Figure S2 and Supplement).

In AD, 3R/4R NFTs arise in medial temporal lobe and locus coeruleus prior to apparent spread to other regions. In PSP, 4R-tau pathology likely begins in globus pallidus and brainstem and spreads via glial and neuronal elements. Whether AD-related 3R/4R-tau species can interact with PSP-related 4R-tau species is unknown [1]. Using immunostaining and RT-QuIC, we observed distributions of PSP-related tauopathy in ADNC + PSPi similar to those reported in primary PSP, consistent with prior case reports. We did not observe alterations in pathological distribution of AD or PSP-specific tau species that would indicate a direct interaction, consistent with other work suggesting that AD and PSP-specific tau species are largely distinct [1]. Our findings are also consistent with prior work showing selectivity of RT-QuIC assays for 3R/4R-tau versus 4R-tau in midfrontal lobe of various tauopathies (see references in Supplement).

Many seeding assays qualitatively indicate seed occurrence and are not isoform-specific, whereas RT-QuIC allows quantification of isoform-specific regional seeding activity over a billion-fold dynamic range. Our work provides the first evidence that tau RT-QuIC can selectively indicate pathologic seed burden in a neuroanatomic fashion, highlighting its potential to interrogate pathophysiology in neurodegenerative diseases. This study is limited by small case numbers, indicative of the rarity of this pathological combination. We also cannot fully differentiate incipient PSP from ‘form-fruste’ PSP: Although cases had yearly structured clinical evaluations and PSP was not clinically evident, emergence of diagnostic symptoms at the end of life cannot be fully excluded (interval from last evaluation to death: 0.5–6 years). Future directions include studying larger cohorts of tauopathies, including ‘pure’ AD, ‘pure’ PSP, and PSP with AD co-pathology, to more precisely delineate the extent to which RT-QuIC can measure isoform-specific tau burden in a neuroanatomic manner.﻿

## Supplementary Information

Below is the link to the electronic supplementary material.

Supplementary file1 (PDF 6206 KB)

## References

[1] Ebashi M (2019). How to demix Alzheimer-type and PSP-type tau lesions out of their mixture-hybrid approach to dissect comorbidity. Acta Neuropathol Commun.

[2] Höglinger GU (2017). Clinical diagnosis of progressive supranuclear palsy: The movement disorder society criteria. Mov Disord.

