# Supplementary material for: Selective tau seeding assays and isoform-specific antibodies define neuroanatomic distribution of progressive supranuclear palsy pathology arising in Alzheimer’s disease
Source: Acta Neuropathol. 2022 Aug 18;144(4):789–92. doi: 10.1007/s00401-022-02480-x (PMC9467964; doi:10.1007/s00401-022-02480-x)
Supplement: Supplementary file 1 — Supplementary file1 (PDF 6206 KB) [file 401_2022_2480_MOESM1_ESM.pdf]

Supplemental Material for:

Selective tau seeding assays and isoform-specific antibodies define neuroanatomic distribution of progressive supranuclear palsy pathology arising in Alzheimer's disease

David G. Coughlin[1], Vanessa S. Goodwill[2#], Heidi G. Standke[3#], Yongya Kim[2], Nicolas Coley[2], Donald P. Pizzo[2], Douglas Galasko[1], Allison Kraus[3]*, Annie Hiniker[2]*

Departments of [1]Neurosciences and [2]Pathology, University of California, San Diego

[3]Department of Pathology, Case Western Reserve University School of Medicine, Cleveland

#Contributed equally

*Contributed equally

Corresponding Authors:

Annie Hiniker, MD, PhD
Department of Pathology
University of California San Diego
9500 Gilman Drive
La Jolla, CA 92093-0612
Phone: (858) 246-5980
e-mail: ahiniker@health.ucsd.edu

Allison Kraus, PhD
Department of Pathology
Case Western Reserve University School of Medicine
2103 Cornell Road
Cleveland, OH 44106
Phone: 216-368-2422
Email:Allison.kraus@case.edu

Supplemental Table 1: Key published studies of AD and PSP co-pathology

	Manuscript	N	Clinical Characteristics	Neuropathology other than PSP	Methods	Conclusions
**Studies of AD with secondary PSP co-pathology**	Milder et al. (1984) Clin Exp. Neurol. PMID: 6568940 [15]	1 case with mixed AD and PSP pathology	Age of onset: 66<br>Age of death: 70<br>Clinical diagnosis: Initially AD, later diagnosed with PSP	AD (not staged)	HE, Holmes silver stain	AD may co-occur with PSP and lead to more severe dementia than typical of PSP
	Sakamoto et al. (2009) Neuropath. PMID: 18992014 [20]	2 cases of mixed AD and PSP pathology	1 male, 1 female<br>Age at death: 80, 82<br>Clinical diagnoses:<br>1 probable AD<br>1 probable PSP	Case 1: Braak tau stage: V, CERAD Amyloid stage C<br>Case 2: Braak tau stage: IV, CERAD Amyloid stage: C	HE-LFB, Bodian stain, Gallyas silver stain, methenamine silver stain	Comorbid PSP and AD pathology complicates diagnosis with a blended phenotypic presentation
	Ebashi M et al. (2019) Acta Neuropath. Commun. PMID: 31060611 [4]	5 cases of mixed AD and PSP pathology (2.8% of 180 cases studied)	4 male, 1 female<br>Mean age at death: 85 (range 80-94)<br>Clinical diagnoses:<br>1 possible PSP<br>1 possible DLB<br>1 AD, 1 CHF, 1 pneumonia	Braak tau: median V (range II-V)<br>CERAD Amyloid stage: median B (range 0-C)<br>2 with AGD, 1 with CAA, 1 with LB	IHC:<br>3R tau (RD3),<br>4R tau (RD4),<br>phospho-tau (AT8)	AD and PSP patterns are conserved in co-pathology cases and can be de-mixed
**Mixed AD and PSP studies**	Gearing et al. (1994) Neurology PMID: 8208392 [5]	13 cases with PSP pathology:<br>4 with PSP + definite AD<br>2 with PSP + probable AD	PSP+definite AD pathology (n=4):<br>Mean age at death: 74.3<br>Mean disease duration: 6.5<br>Mean duration of dementia: 4.5<br><br>Clinical diagnoses: PSP+AD (probable or definite, n=6): 4 PSP, 1 AD, 1 AD and PD	No case level data on pathological findings	HE, Bielschowsky staining, thioflavin S, IHC: ubiquitin, amyloid-beta (10D5)	Co-pathologies within PSP are common and contribute to clinical heterogeneity
	Urasaki et al. (2000) Neuropathol. PMID: 11132941 [21]	1 case with mixed AD and PSP pathology	Age at symptom onset: 69<br>Age at death: 79<br>Clinical diagnosis: PSP	AD per CERAD criteria<br>Braak stage V<br>CAA	HE, Kluever-Barrera, Bodian, Bielschowsky, modified Gallyas-Braak, IHC: tau and beta-amyloid	PSP and AD co-occurrence is unexpectedly rare and may represent a subgroup of tauopathy
	Yoshida et al. (2017) Acta Neuropathol. PMID: 28064358 [23]	29 cases of 'incipient' PSP (2.9% of 998 cases examined)	11 male, 18 female<br>Mean Age at death: 82.3 (range 64-94)<br>Clinical diagnoses:<br>3 PD<br>2 LBD	Braak tau: median III (range 0-VI)<br>CERAD Amyloid stage: median A (range 0-C)<br>Low NIA-AA: 14<br>High NIA-AA: 1<br>AGD: 21<br>LB: 8 $\geq$ III/VI<br>TDP-43: 12<br>ARTAG: 28	HE, Gallyas silver staining<br>IHC:<br>phospho-tau (AT8)<br>3R tau (RD3)<br>4R tau (RD4)<br>αSyn (LB509)<br>TDP-43<br>amyloid beta (6F/3D)	Incipient PSP cases may be more common than previously thought. Only some had co-ocurring AD pathology. High rates of ARTAG were noted

	Dugger et al. (2014) Parkinsonism and Relat Disord. PMID: 24637124 [3]	64 cases of PSP examined for co-pathologies	41 male, 23 female Mean age at death 84 (SD 8.5) Disease duration: 8 (SD 6.2)	36% had AD, 21% LB, 44% AGD, 25% CAA	HE, Gallyas, Thioflavin-S, Campbell-Switzer, IHC: αSyn	Co-pathologies in PSP are common
**Studies of PSP with secondary AD co-pathology**	Keith-Rokosh & Ang (2008) Can J Neurol Sci. PMID: 19235444 [9]	32 cases with PSP neuropathology were examined for co-pathologies	19 male, 13 female Mean age at death: 73.35 (range 55-85) Disease duration: Median 5.22 years (range 1.5-11) Clinical diagnoses: 15 PSP, 5 AD, 2 vascular dementia, 2 'functional decline', 1 Pick's, 1 PD/AD, 1 PD, 1 ALS, 1 cerebellar degeneration, 1 PPA	AD related changes in 69% (Braak 1-5), six (18.75%) with probable or definite AD per CERAD criteria 10 cases with overlapping 'CBD' features 4 cases with Lewy bodies 5 cases with infarcts 7 cases with 'pure' PSP pathology	HE, HE/LFB, Bielschowsky, Congo red, IHC: αSyn phospho-tau (AT8) GFAP	Co-pathologies, specifically AD, are frequent with PSP and may play a role in the development of cognitive dysfunction in PSP patients
	Robinson et al. (2018) Brain PMID: 29878075 [18]	51 of 766 brains examined assigned a primary diagnosis of PSP	Mean age of onset: 68 (SD 8) Disease duration: 8 (SD 4) Age at death: 76 (SD 8)	57% with amyloid beta 22% with alpha-synuclein 12% with neocortical TDP-43	HE, tau, amyloid beta (or Thioflavin S), alpha-synuclein, TDP-43	PSP has a high rate of ADNC co-pathology. Only cases with co-pathologies were APOE Ɛ4 positive
	Jecmenica-Lukic et al. (2020) Mov Disord PMID: 32125724 [8]	101 PSP patients assessed for co-pathologies	57 male, 44 female Age of onset 65.5 (SD 7.9) Age at death: 73.1 (SD 7.0) Disease duration 7.6 (SD 4.2)	84 cases with some degree of AD pathology, 54 with both RD3 and Aβ pathology but none reaching "intermediate" or "high" ADNC using this criteria	HE IHC: αSyn phospho-tau (AT8) Aβ (4G8) 3R tau (RD3) TDP43 (409/410) FUS	Age at onset and age at death were associated with higher rates of co-pathology, but co-pathology was not related to major disease milestones

Abbreviations: AGD: argyrophilic grain disease, ARTAG: aging-related tau astrogliopathy, CHF: congestive heart failure, IHC: Immunohistochemistry, HE: hematoxylin and eosin, HE-LFB:hematoxylin and eosin with luxol fast blue, LB: Lewy body, NIA-AA: National institute on Aging-Alzheimer's Association criteria, PD: Parkinson's disease, AD: Alzheimer's disease, PSP: Progressive supranuclear palsy, PPA: Primary progressive aphasia, SD: standard deviation

Supplemental Methods

Case selection: 1296 individuals autopsied at the UCSD Shiley-Marcos Alzheimer's Disease Research Center (ADRC) met criteria for intermediate or high degree Alzheimer's Disease Neuropathologic Change (ADNC) according to NIA-AA consensus criteria (i.e. levels of ADNC sufficient to explain a dementia syndrome) [16]. Using this cohort, the UCSD ADRC neuropathology database was screened for a secondary diagnosis of progressive supranuclear palsy (PSP), resulting in the 5 cases included in this study. For this study, we were interested specifically in cases with intermediate or high ADNC with more limited PSP pathology and thus excluded cases with a primary pathologic diagnosis of PSP who had AD co-pathology (N = 10). To determine if observed seeding was an age-related phenomenon or related to tau pathology accumulations, two cases of cognitively normal older adults with low Braak tau stage (I/VI) and two cases of Huntington's disease who died in their 30s (Braak 0/VI) were selected as control cases (Supplemental Table 2).

Supplemental Table 2: Control Case Characteristics

	Control Cases:			
**Characteristics:**	**6**	**7**	**8**	**9**
**Sex**	Female	Male	Male	Male
**Age at Death**	35	37	75	84
**Disease Duration (y)**	20	Not recorded	NA	NA
**Clinical Diagnosis**	Huntington's Disease	Huntington's Disease	Normal	Normal
**Brain Weight (g)**	958	1140	1216	1188
**Thal Phase**	A0	A1	A0	A0
**Braak Stage**	0, B0	0, B0	I, B1	I, B1
**CERAD Score**	C0	C0	C0	C0
**ADNC**	Not	Low	Not	Not
**LBD Stage**	None	None	None	None
**Other**	Huntington's disease	Huntington's disease	Mild Arteriosclerosis	Acute ischemic changes

Diagnostic neuropathologic evaluation: Autopsy was performed using a previously described protocol [1]. Brains were divided sagittally and the left hemibrain was fixed in 10% buffered formalin. Right hemibrain was frozen at -80°C for future biochemical studies. After at least 14 days, the formalin-fixed hemibrain was serially sectioned into 1 cm slices. Sections were taken, paraffin-embedded, and stained with hematoxylin and eosin (H&E) for histopathological examination including middle frontal cortex (Brodmann areas 8/9), rostral superior temporal cortex, inferior parietal cortex, hippocampus (CA1-CA4 and dentate gyrus), entorhinal cortex, basal ganglia, midbrain with substantia nigra, pons with locus coeruleus, and cerebellar cortex with dentate nucleus.

Neuritic and diffuse plaques were identified either with 1% thioflavin-S stain on 10 µm-thick sections viewed with ultraviolet illumination and a 440 µm bandpass wavelength excitation filter, or with immunohistochemical (IHC) staining using antibodies to Amyloid beta (Aβ) (Ab 69D, rabbit polyclonal from Edward Koo, 1:1200). Tau pathology was visualized using paired helical filament (PHF) tau antibody (PHF1 from Peter Davies, 1:600) on 5 µm-thick sections. Pathological diagnosis of AD was made using NIA-AA consensus criteria [16]. PSP pathology was identified using characteristic morphologic features including tufted astrocytes, globose neurofibrillary tangles, and oligodendroglial coiled bodies [7, 10].

Lewy pathology was identified by characteristic morphology and immunostaining for α-synuclein (phospho-synuclein 81A, from Virginia Lee, 1:15,000) and staged according to consensus Dementia with Lewy Bodies (DLB) guidelines [13]. TDP-43 neuropathology (TDP-43 from Proteintech #10782-2-AP, 1:12,000) was evaluated in hippocampus and middle frontal gyrus only for cases 1-4 (due to absence of amygdala block for these cases) and using full Limbic-predominant Age-related TDP-43 Encephalopathy (LATE) consensus guidelines for case 5 [17]. Hippocampal sclerosis (HS) was diagnosed independent of TDP-43 pathology when neuronal loss in the CA1 and subiculum was out of proportion with the degree of AD pathology. Vascular pathology was evaluated using gross assessment for large and lacunar infarcts,

atherosclerosis of the circle of Willis, and hemorrhage, and microscopic evaluation for arteriolosclerosis, microhemorrhages, and amyloid angiopathy.

Tau isoform immunohistochemistry: 5-6 μM tissue sections were cut from formalin-fixed paraffin-embedded human brain tissue and mounted onto slides. Slides were stained on a Ventana Discovery Ultra (Ventana Medical Systems, Tucson, AZ, USA) with antibodies to AT8 (phospho-Ser202/Thr305 tau, Thermo MN1020; 1:300), 3R tau (RD3, Invitrogen; 05-803, 1:300), 4R tau (RD4, Sigma; 05-804 1:750), and GT38 (AD-specific tau, Abcam; ab246808, 1:300) [6]. No antigen retrieval was needed for AT8 immunohistochemistry. For other epitopes the optimal retrieval was determined independently. For both RD3 and RD4 antigen, retrieval consisted of treatment with protease 2 (Ventana Medical systems) for 20 min at 37°C followed by 64 min with CC1 (Tris-EDTA; pH 8.6) at 95°C. For GT38 antigen, retrieval with CC1 was done for 40 min at 95°C. Primary antibodies were incubated on the slides for 32 minutes at 37°C followed by treatment with goat anti-mouse HRP-polymer secondary (prediluted from Ventana Medical systems) at 37°C for 12 minutes**.** For single epitope immunohistochemistry, antibody presence was visualized using DAB as a chromagen followed by hematoxylin counterstain. Slides were rinsed in Dawn detergent (to remove the liquid coverslip oil) in water, dehydrated with alcohol and xylene and coverslipped.

Immunofluorescence: For dual RD4/GT38 staining, RD4 antibody was incubated on the tissue as above followed by incubation with HRP-labeled goat anti-mouse antibody (prediluted from Ventana Medical systems) then detected using TSA Alexa-fluor 488 (Invitrogen; diluted 1:100 and incubated at 37°C for 20 min). Subsequently, sections were incubated in CC2 (Ventana Medical Systems; citrate; pH 6) and heated to 95°C for 24 min to strip away all antibodies (as validated for this system for clinical use). The sections were then incubated with the GT38 antibody (as above) followed by incubation with HRP-labeled goat anti-mouse (as above) and

detected using TSA-Alexa 594 (Invitrogen; diluted 1:100 and incubated at 37°C for 20 min). Sections were rinsed in Dawn detergent (to remove the liquid coverslip oil) in water and coverslipped with Vectashield containing DAPI (VectorLabs) and imaged on a Leica DMi8 microscope with a 60x oil objective.

Semi-quantitative analysis of immunohistochemistry: Pathological tau accumulations (GT38, RD3, RD4, and AT8) in each region were rated by two neuropathologists (AH and VG) and a neurologist trained in neuropathology (DC) on a scale from 0-3 (0:none, 1:mild, 2:moderate, and 3:severe). AT8-positive pathology was classified as astrocytic, oligodendroglial, or neuronal. Raters were blinded to RT-QuIC seeding activity values and to clinical history. To establish the upper range of pathological inclusions for semi-quantitative scoring, regions with abundant pathology from positive control cases were utilized to define '3: severe' degree of pathological inclusions. These regions included entorhinal cortex/hippocampus from AD with NIA-AA criteria “high” ADNC for GT38 and RD3-positive pathology and AT8-positive neuronal pathology, pons from PSP without AD pathology for RD4-positive pathology; and striatum from PSP without AD pathology for AT8-positive astroglial pathology and AT8-positive oligodendroglial pathology. Low and intermediate levels of tau inclusions were estimated by raters at their discretion. Raters performed their ratings independently and then ratings were compared, if there was a discrepancy between raters, the slide was evaluated jointly and a consensus was reached. Comparisons of severity of pathological inclusions (0-3 scores) identified by different tau isoform specific antibodies (RD4, RD3, GT38) were done in the globus pallidus, entorhinal cortex, and middle frontal cortex using Wilcoxon rank sum tests.

RT-QuIC Methods:

Tissue homogenization

10% w/v brain tissue homogenates were prepared from the dissected brain tissue regions in ice-cold 1x PBS with cOmplete EDTA-free protease inhibitors (Roche) and homogenized using 1 mm zirconia/silica beads (BioSpec Products 11079110z) in a BeadMill. Homogenates were placed on ice for 5 minutes before centrifugation at 2,000g for 2 minutes. Supernatant was collected, aliquoted and used immediately or stored at -80°C until RT-QuIC analysis.

RT-QuIC analysis

Tau RT-QuIC assays to quantify 4R and 3R/4R tau seeds were performed based on previously published protocols, with 3-5 independent endpoint dilution analyses for cases 1 – 5, and at least two replicates for comparative controls [cases 6-9 and mouse tau knockout (KO)] as indicated in the figures [14, 19]. For case 1-5, three replicates were completed for each case, with additional replicates if inter-assay variance for a determined SD_{50} was greater than >1 log. Tau recombinant protein substrates were purified and used for RT-QuIC analysis of brain homogenates. Endpoint dilution analysis (quadruplicate wells at each dilution) was conducted with dilution of homogenates in diluent buffer (0.526% mouse tau KO brain homogenate/N2/10mM Hepes pH 7.4) with dilutions used to seed 4R RT-QuIC (7.5 µM K18CFh and 3.75 µM K19CFh recombinant tau, 90 µM poly-glutamate [Sigma, P1818], 40 mM HEPES pH 7.4, 200 mM sodium citrate, and 10 µM thioflavin T [ThT] with one zirconia/silica bead) and K12 (3R/4R) RT-QuIC (6.5 µM K12CFh, 40 µM heparin, 40 mM HEPES, 400 mM NaF, and 10 µM ThT) in a 384-well optical plate (Thermo Scientific Nunc, 242764). Plates were sealed (Nunc 232702) and incubated at 42°C or 30 °C, for K12 or 4R RT-QuIC respectively with alternate 1-minute cycles of orbital shaking (500 rpm) and rest in a BMG FLUOstar Omega plate reader. Fluorescence reads were taken every ~45 minutes, bottom read, using 450-10 nm excitation and 480-10 nm emission.

Spearman Kärber

Endpoint dilution analysis was conducted where indicated and per Spearman-Kärber [2] used to determine seeding doses or SD_{50}s as the dilution at which 50% of quadruplicate wells are positive as previously described [12, 22]. Positive wells are determined by ThT fluorescent values that exceed a threshold of five consecutive baseline reads of control (mouse tau KO) + 100 x standard deviation.

Statistical Analysis of RT-QuIC:

Statistical analysis was conducted as indicated using GraphPrism 9.1.2. Seeding doses are shown as $\log_{10}$ values. P values were determined using one-way ANOVA to compare seeding doses (SD_{50}s) between regions and cases. For 3R/4R versus 4R region-specific seeding dose comparisons as reported in the main text (Fig. 1c, $p<0.0001$), log differences in seeding activities were calculated using a reference value determined by the lowest average SD_{50} of all cases and regions determined for each assay, and those values used to determine comparative log differences of 4R from 3R/4R seeding activities. Log differences were averaged across the independent replicates of the four ADNC+PSPi cases analyzed (Case 1, 2, 4, 5) and used for one-way ANOVA with Tukey's post-hoc multiple comparisons test to determine p values.

Supplemental Results

GT38 and RD4 dual immunofluorescence: We expanded the dual immunofluorescence neuroanatomic analysis in Figure 1a and performed dual immunofluorescence with GT38 and RD4 on midfrontal lobe, hippocampus, and globus pallidus of all five ADNC + PSPi cases (Figure S1). In keeping with our semiquantitative analyses (Figure 1b), this demonstrated consistent region-specific pathology among all five cases, with frequent GT38/RD4 double positive neurofibrillary tangles (NFTs) in midfrontal cortex and hippocampus and RD4-positive/GT38 negative astrocytes and oligodendroglia prominent in basal ganglia.

Semiquantitative evaluation of tau isoform regional abundance: For semiquantitative comparisons of anatomic regional abundance of pathology (Figure 1b), abundance of pathologic inclusions (0-3 scores) identified by different tau isoform-specific antibodies (RD4, RD3, GT38) was compared across cases in the globus pallidus, hippocampus/entorhinal cortex, and middle frontal cortex using Wilcoxon rank sum tests. In the globus pallidus, RD4-positive inclusions were more severe than RD3-positive or GT38-positive inclusions (z=2.6, 2.8 and p=0.009, 0.005 respectively). In the hippocampus/entorhinal cortex and middle frontal cortex, the severity of RD4-positive inclusions was similar to severity of RD3-positive and GT38-positive inclusions (p>0.05). In the hippocampus/entorhinal cortex, this likely represents primarily AD pathology, whereas in the middle frontal cortex, this likely represents a mix of AD and PSP pathology.

Table S3: Semiquantitative comparative severity of inclusions

**Region**	**RD4**	**RD3**	**GT38**	**RD4 v RD3**	**RD4 v GT38**
Globus Pallidus	2 [2,2]	0 [0,0]	0 [0,0]	Z=2.6 p=0.009	Z=2.9 p=0.005
Entorhinal Cortex	3 [3,3]	3 [3,3]	3 [3,3]	Z=incalculable, p=incalculable	Z=incalculable, p= incalculable
Middle Frontal Cortex	1 [1,2]	0 [0,1]	1 [1,2]	Z=1.4 p=0.15	Z=0.0 p=1.0

Data shown are median and interquartile range

Seeding Characteristics in Control Cases: Brain regions obtained from two cases of Huntington's disease or two cases of Braak tau stage I (see Supplemental Table 2 for patient characteristics) demonstrated significantly less 3R/4R and no 4R tau seeding activity when compared to AD/PSP cases 1 – 5 (Figure S2). Representative RT-QuIC readouts are shown for endpoint dilution analysis for the brain region indicated for two cases with Huntington's disease, and two cognitively normal cases of Braak I neuropathology. Higher than anticipated 3R/4R tau seeds are noted in the globus pallidus of Braak 1 controls, however, ultrasensitive RT-QuIC methodology has previously indicated seeds without histological tau identification [11].

Importantly, no seeding doses are detectable in mouse tau knock-out (mKO) brain homogenate (BH) as a negative control. Figure S3 and S4 indicate representative endpoint RT-QuIC analysis, where each curve represents fluorescence readouts (arbitrary units) of an individual of quadruplicate wells seeded at each brain tissue dilution. SD_{50} values calculated from endpoint dilution analysis are as shown with asterisks indicating calculatable mathematical values of assay baseline that indicate no seeding activity was detectable at 10^{-3} brain homogenate dilution.

Supplemental Figures and Legends:

Figure S1 (next page). Immunofluorescence images from all cases stained for GT38 (red) and RD4 (green) with a DAPI (blue) counterstain from the middle frontal cortex, basal ganglia, and hippocampus. The hippocampus shows a large number of neurofibrillary tangles which stain positive for both GT38 and RD4, the basal ganglia shows a high degree of astroglial pathology that stains for RD4 but not GT38 and the middle frontal cortex contains a mixture of AD elements (staining positive for both GT38 and RD4) and PSP related elements (staining positive for RD4 alone). Images taken using a Leica DMi8 microscope at 40x with scale bar of 25µm. Antibodies utilized were AT8 (MN1020, Thermo 1:300), 3R tau (8E6/C11, RD3 Invitrogen, 1:750), 4R tau (7D12.1 RD4, Sigma Aldrich 1:300), and AD-specific tau (GT38, Abcam 1:750).

Figure S1

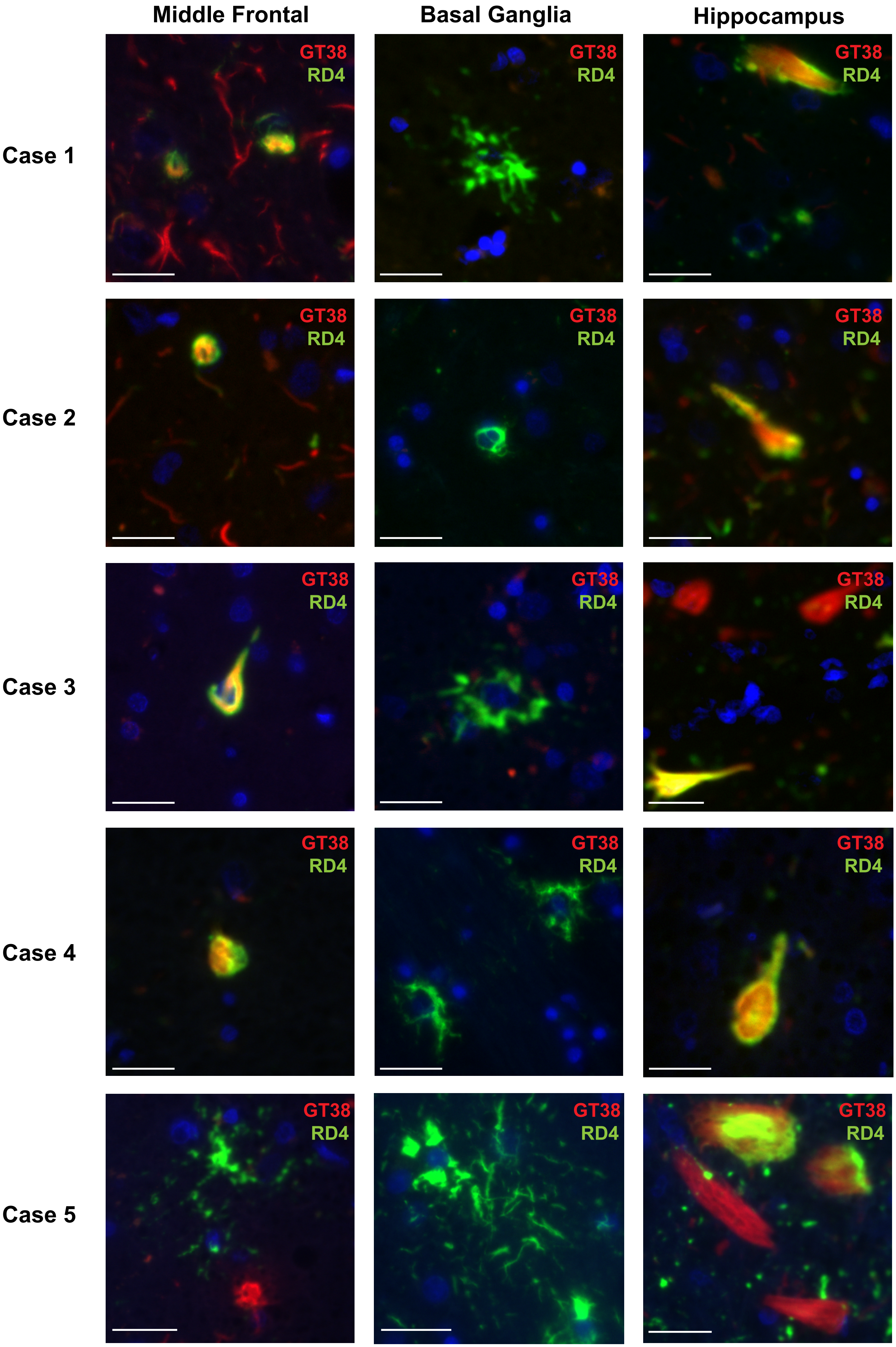
Middle Frontal
Basal Ganglia
Hippocampus
Case 1
Case 2
Case 3
Case 4
Case 5
GT38
RD4

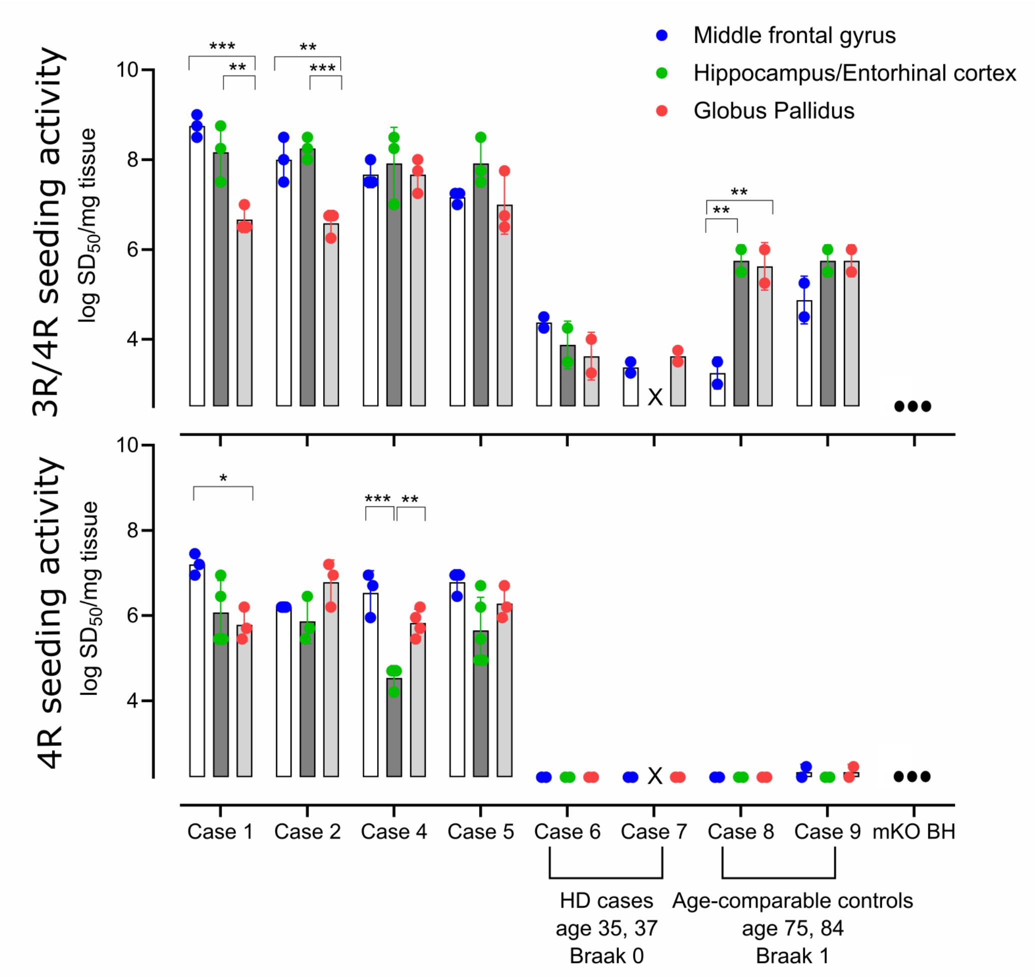

Figure S2: Regional seeding doses of ADNC + PSPi and control cases determined using 3R/4R or 4R selective RT-QuIC assays. Seeding doses on a log 10 scale (log SD_{50}) are shown for each case and region. Each data point indicates an independent SD_{50} determination, with 2-5 replicates for each of three regions (middle frontal gyrus [MF], hippocampus/entorhinal cortex [HP/EC], globus pallidus [GP]) per case. Average seeding doses and standard deviation are indicated by bar and standard error bars. There were significant differences in 3R/4R seeding between the sample cases (case 1-5) and control cases (cases 6-9) per region (MF p=0.0003, HP/EC p=0.0027, and GP p=0.0113). All 3R/4R seeding doses and those 4R seeding doses determined for Case 1-5 had significantly more seeding activity ($p<0.0001$) when compared to mouse tau knockout. “x” indicates region not available. HD, Huntington’s disease; mKO BH, mouse tau knockout brain homogenate. *$p<0.05$, **$p<0.01$, ***$p<0.001$.

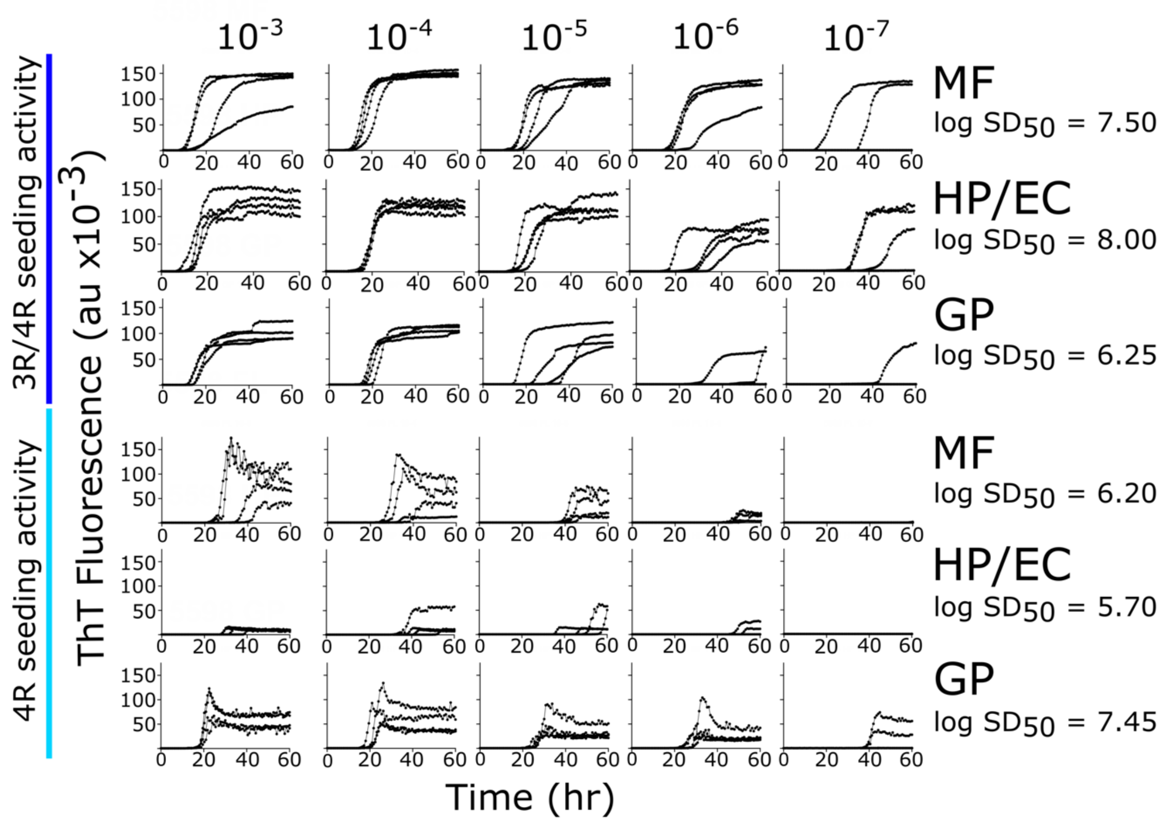

Figure S3: Seeding Characteristics of an AD+PSPi case: Endpoint dilution analysis of brain regions in 3R/4R (K12) and 4R tau RT-QuIC assays. Representative RT-QuIC readouts are shown for Case 2 endpoint dilution analysis for the brain region indicated. Each curve represents fluorescence readouts (arbitrary units, au) of an individual of a total of quadruplicate wells seeded at each brain tissue dilution. Some brain regions required additional dilutions not shown here (i.e. 10^{-8} and 10^{-9}) to reach endpoint. Log SD_{50} values calculated from endpoint dilution analysis are as shown with all designated positive wells exceeding a threshold of average baseline readings + 100x standard deviation. MF, middle frontal gyrus; HP/EC, hippocampus/entorhinal cortex; GP, globus pallidus.

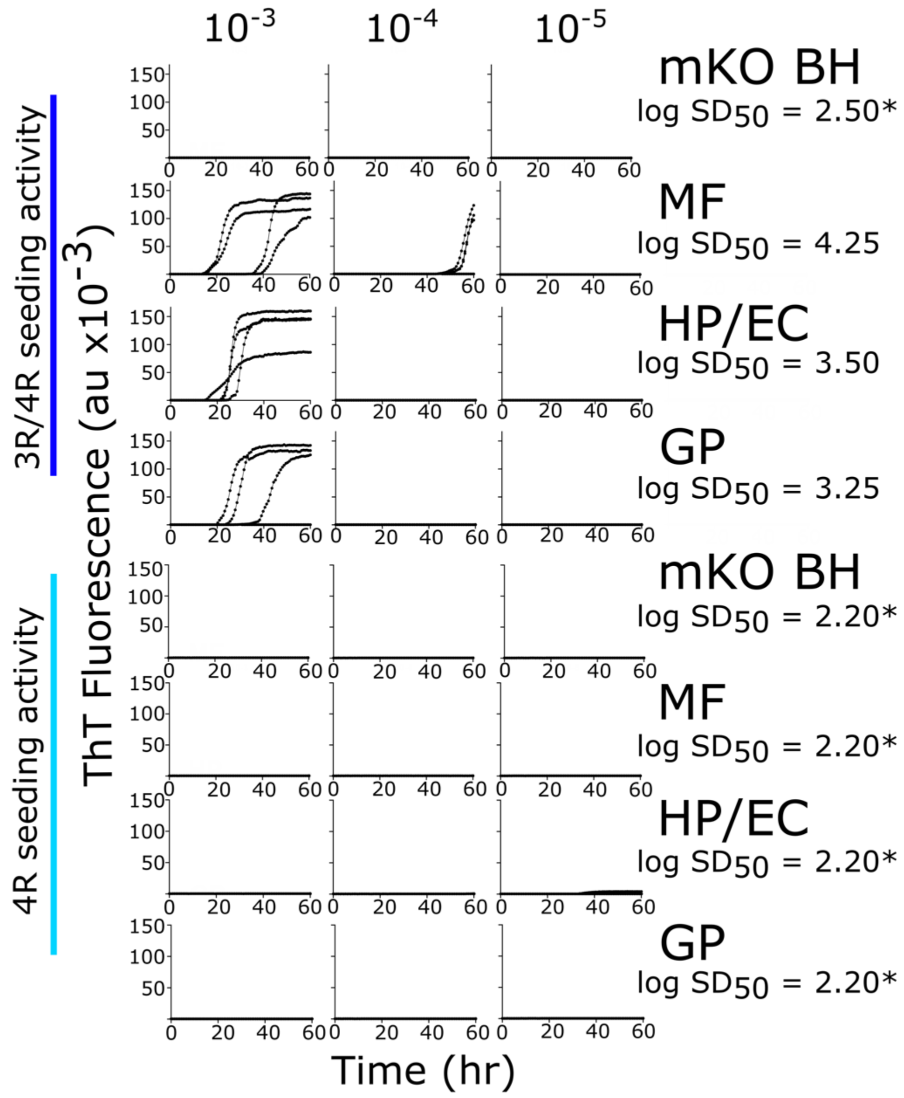

Figure S4: Seeding Characteristics of a Control Case and mouse tau knockout: Endpoint dilution analysis of brain regions in 3R/4R (K12) and 4R tau RT-QuIC assays. Representative RT-QuIC readouts are shown for mouse tau knockout brain homogenate and control Case 6 for the brain region indicated. Each curve represents fluorescence readouts (arbitrary units, au) of a single well of quadruplicate wells seeded at each brain tissue dilution. Log SD_{50} values calculated from endpoint dilution analysis are as shown on the right. Asterisks indicate log SD_{50} values of assay baseline, i.e. no seeding activity detected at minimum required 10^{-3} brain tissue homogenate dilution. mKO BH, mouse tau knockout brain homogenate; MF, middle frontal gyrus; HP/EC, hippocampus/entorhinal cortex; GP, globus pallidus.

Supplemental References:

1. Adamowicz DH, Roy S, Salmon DP, Galasko DR, Hansen LA, Masliah E, Gage FH (2017) Hippocampal alpha-Synuclein in Dementia with Lewy Bodies Contributes to Memory Impairment and Is Consistent with Spread of Pathology. J Neurosci 37:1675–1684. doi: 10.1523/jneurosci.3047-16.2016
2. Dougherty R (1964) Animal virus titration techniques. Academic Press, New York
3. Dugger BN, Adler CH, Shill HA, Caviness J, Jacobson S, Driver-Dunckley E, Beach TG (2014) Concomitant pathologies among a spectrum of parkinsonian disorders. Parkinsonism Relat Disord 20:525–529. doi: 10.1016/J.PARKRELDIS.2014.02.012
4. Ebashi M, Ito Y, Uematsu M, Nakamura A, Hirokawa K, Kamei S, Uchihara T (2019) How to demix Alzheimer-type and PSP-type tau lesions out of their mixture -hybrid approach to dissect comorbidity. Acta Neuropathol Commun 7. doi: 10.1186/s40478-019-0708-4
5. Gearing M, Olson DA, Watts RL, Mirra SS (1994) Progressive supranuclear palsy: neuropathologic and clinical heterogeneity. Neurology 44:1015–1024. doi: 10.1212/WNL.44.6.1015
6. Gibbons GS, Kim SJ, Robinson JL, Changolkar L, Irwin DJ, Shaw LM, Lee VMY, Trojanowski JQ (2019) Detection of Alzheimer's disease (AD) specific tau pathology with conformation-selective anti-tau monoclonal antibody in co-morbid frontotemporal lobar degeneration-tau (FTLD-tau). Acta Neuropathol Commun 7:34. doi: 10.1186/s40478-019-0687-5
7. Hauw J-J, Daniel SE, Dickson D, Horoupian DS, Jellinger K, Lantos PL, McKee A, Tabaton M, Litvan I (1994) Preliminary NINDS neuropathologic criteria for Steele-Richardson-Olszewski syndrome (progressive supranuclear palsy). Neurology 44:2015
8. Jecmenica Lukic M, Kurz C, Respondek G, Grau-Rivera O, Compta Y, Gelpi E, Troakes C, Barcelona Brain Bank collaborative group the MPSP study group, van Swieten JC, Giese A (2020) Copathology in Progressive Supranuclear Palsy: Does It Matter? Mov Disord 35:984–993
9. Keith-Rokosh J, Ang LC (2008) Progressive supranuclear palsy: a review of co-existing neurodegeneration. Can J Neurol Sci 35:602–608. doi: 10.1017/S0317167100009392
10. Kovacs G, Lukic MJ, Irwin DJ, Arzberger T, Respondek G, Lee EB, Coughlin D, Giese A, Grossman M, Kurz C (2020) Distribution patterns of tau pathology in progressive supranuclear palsy. Acta Neuropathol
11. Kraus A, Saijo E, Metrick 2nd MA, Newell K, Sigurdson CJ, Zanusso G, Ghetti B, Caughey B (2019) Seeding selectivity and ultrasensitive detection of tau aggregate conformers of Alzheimer disease. Acta Neuropathol 137:585–598. doi: 10.1007/s00401-018-1947-3
12. Kraus A, Saijo E, Metrick MA, Newell K, Sigurdson CJ, Zanusso G, Ghetti B, Caughey B (2019) Seeding selectivity and ultrasensitive detection of tau aggregate conformers of Alzheimer disease. Acta Neuropathol 137:585–598. doi: 10.1007/S00401-018-1947-3
13. McKeith IG, Boeve BF, Dickson DW, Halliday G, Taylor J-P, Weintraub D, Aarsland D, Galvin J, Attems J, Ballard CG (2017) Diagnosis and management of dementia with Lewy bodies Fourth consensus report of the DLB Consortium. Neurology 10.1212/WNL. 0000000000004058
14. Metrick MA, Ferreira NDC, Saijo E, Kraus A, Newell K, Zanusso G, Vendruscolo M, Ghetti B, Caughey B (2020) A single ultrasensitive assay for detection and discrimination of tau aggregates of Alzheimer and Pick diseases. Acta Neuropathol Commun 8. doi: 10.1186/S40478-020-0887-Z
15. Milder DG, Elliott CF, Evans WA (1984) Neuropathological findings in a case of

coexistent progressive supranuclear palsy and Alzheimer's disease. Clin Exp Neurol 20:181–187
16. Montine TJ, Phelps CH, Beach TG, Bigio EH, Cairns NJ, Dickson DW, Duyckaerts C, Frosch MP, Masliah E, Mirra SS, Nelson PT, Schneider JA, Thal DR, Trojanowski JQ, Vinters H V, Hyman BT (2012) National Institute on Aging-Alzheimer's Association guidelines for the neuropathologic assessment of Alzheimer's disease: a practical approach. Acta Neuropathol 123:1–11. doi: 10.1007/s00401-011-0910-3
17. Nelson PT, Dickson DW, Trojanowski JQ, Jack CR, Boyle PA, Arfanakis K, Rademakers R, Alafuzoff I, Attems J, Brayne C, Coyle-Gilchrist ITS, Chui HC, Fardo DW, Flanagan ME, Halliday G, Hokkanen SRK, Hunter S, Jicha GA, Katsumata Y, Kawas CH, Keene CD, Kovacs GG, Kukull WA, Levey AI, Makkinejad N, Montine TJ, Murayama S, Murray ME, Nag S, Rissman RA, Seeley WW, Sperling RA, White CL, Yu L, Schneider JA (2019) Limbic-predominant age-related TDP-43 encephalopathy (LATE): Consensus working group report. Brain 142:1503–1527
18. Robinson JL, Lee EB, Xie SX, Rennert L, Suh E, Bredenberg C, Caswell C, Van Deerlin VM, Yan N, Yousef A (2018) Neurodegenerative disease concomitant proteinopathies are prevalent, age-related and APOE4-associated. Brain
19. Saijo E, Metrick MA, Koga S, Parchi P, Litvan I, Spina S, Boxer A, Rojas JC, Galasko D, Kraus A, Rossi M, Newell K, Zanusso G, Grinberg LT, Seeley WW, Ghetti B, Dickson DW, Caughey B (2020) 4-Repeat tau seeds and templating subtypes as brain and CSF biomarkers of frontotemporal lobar degeneration. Acta Neuropathol 139:63. doi: 10.1007/S00401-019-02080-2
20. Sakamoto R, Tsuchiya K, Yoshida R, Itoh Y, Furuta N, Kosuga A, Sugai Y, Mimura M (2009) Progressive supranuclear palsy combined with Alzheimer's disease: A clinicopathological study of two autopsy cases. Neuropathology 29. doi: 10.1111/j.1440-1789.2008.00968.x
21. Urasaki K, Kuriki K, Namerikawa M, Satoh S, Ikeguchi K, Saito K, Fukayama M, Nakano I (2000) An autopsy case of Alzheimer's disease with a progressive supranuclear palsy overlap. Neuropathology 20. doi: 10.1046/j.1440-1789.2000.00334.x
22. Wilham JM, Orrú CD, Bessen RA, Atarashi R, Sano K, Race B, Meade-White KD, Taubner LM, Timmes A, Caughey B (2010) Rapid End-Point Quantitation of Prion Seeding Activity with Sensitivity Comparable to Bioassays. PLOS Pathog 6:e1001217. doi: 10.1371/JOURNAL.PPAT.1001217
23. Yoshida K, Hata Y, Kinoshita K, Takashima S, Tanaka K, Nishida N (2017) Incipient progressive supranuclear palsy is more common than expected and may comprise clinicopathological subtypes: a forensic autopsy series. Acta Neuropathol 133:809–823. doi: 10.1007/S00401-016-1665-7
